# Gender-based discrimination and its influence on mental health symptoms among people living with and without migraine: A case-control study

**DOI:** 10.1177/13591053251317069

**Published:** 2025-02-19

**Authors:** Venezya H Thorsteinson, Kelsey M Haczkewicz, Natasha L Gallant

**Affiliations:** University of Regina, Canada

**Keywords:** anxiety, depression, discrimination, migraine, trauma, women

## Abstract

Women are more likely than men to experience migraine and to endorse worse symptoms. Migraine is associated with anxiety, depressive and posttraumatic stress disorders. Women who experience migraine are also more likely to report a history of discriminatory experiences. This study investigated migraine characteristics, mental health outcomes and gender-based discrimination among women using a case-control study with a migraine and non-migraine sample. Two hundred ninety-two women completed an online survey with measures of migraine characteristics (as applicable), mental health symptoms, and gender-based discrimination. Women living with migraine experienced worse mental health symptoms and more gender-based discrimination than the non-migraine group. Migraine frequency and lifetime day-to-day discrimination significantly predicted anxiety, depression, and trauma symptoms, while anticipated discrimination significantly predicted trauma symptoms; lifetime day-to-day discrimination significantly predicted migraine-related reduction in productivity; and gender-based discrimination significantly predicted migraine-related social absences. These findings may be used to improve management of migraine among women.

## Introduction

Migraine is a prevalent disorder that affects approximately 11% of adults worldwide (Walter, 2022; World Health Organization, 2011). Previous research suggests that many individuals who live with migraine disorder experience some level of migraine disability (Chu et al., 2018; Renjith et al., 2016). Migraine disability is a condition which limits a person’s ability to complete daily tasks and/or experience a high quality of life due to migraine (Lipton et al., 2003; Renjith et al., 2016). Moderate to severe disability is more commonly experienced than little to mild disability among people living with migraine (Renjith et al., 2016). Common impacts of migraine disability include missing out on school, work, or social events; trouble completing housework; and reduced productivity at school or work. For example, individuals experiencing headache disorders, such as migraine, often experience a substantial loss of paid work hours and reduced work efficiency, which imposes a great burden on the individual (World Health Organization, 2016).

Women are three times more likely than men to experience migraine and tend to endure worse migraine characteristics (Amiri et al., 2022; Vetvik and MacGregor, 2017; World Health Organization, 2011). Globally, migraine is ranked the second cause of years lived with disability for all ages and genders (Steiner et al., 2020). Among women, migraine is the second most significant level-4 factor of global disability, beneath low back pain (Steiner et al., 2020). Further, among young women, migraine is ranked the top cause of disability-adjusted life years (Steiner et al., 2020). Women are also more likely than men to endure increased migraine-related disability symptoms including longer and more intense migraine attacks and migraine-associated activity restriction (Goldstein et al., 2019; Vetvik and MacGregor, 2017). Psychiatric disorders, including anxiety, depression, and PTSD are also more prevalent among people living with migraine (de Leeuw et al., 2005; Fuller-Thomson et al., 2017; McWilliams et al., 2004; Molgat and Patten, 2005). Furthermore, women are also more likely to experience anxiety, depression, and posttraumatic stress disorder (PTSD), than men (American Psychiatric Association, 2020; Farhood et al., 2018; McLean et al., 2011). While the relationship between PTSD and migraine is not well understood, symptoms related to PTSD have the potential to exacerbate disability among migraine populations (Peterlin et al., 2011). Moreover, many individuals who experience migraine comorbid with anxiety or depression report greater migraine frequency (Lipton et al., 2020).

As a result of their gender identity, many women experience discrimination (i.e. the deliberate choice to treat a person or group unjustly specifically due to their personal characteristics) within their workplace and private life (Klonoff and Landrine, 1995; Statistics Canada, 2023). Specifically, women often earn less, face lower promotion rates, and more frequently experience violence (e.g. sexual assault, homicide, & childhood maltreatment) or harassment (e.g. inappropriate sexual behaviors) than men (Statistics Canada, 2023). Discrimination and the anticipation of possible future discrimination can negatively impact an individual’s overall health and wellbeing (American Psychological Association, 2019; Earnshaw et al., 2016; Hua et al., 2023). Rosendale et al. (2022) found that individuals living with migraine often reported a history of discriminatory experiences (e.g. harassment or unequitable treatment). Furthermore, individuals who have experienced both a traumatic event and discrimination reported higher levels of migraine disability compared to individuals who have not (Rosendale et al., 2022). However, current literature is limited in terms of what is known about the influence of gender-based discrimination alone on migraine characteristics and associated mental health symptoms.

The present study therefore aimed to investigate the relationship between migraine characteristics, anxiety symptoms, depressive symptoms, trauma symptoms, and gender-based discrimination among women. Specifically, this study aimed to answer three research questions: (1) Are levels of mental health symptoms and gender-based discrimination significantly different in the migraine versus non-migraine sample? (2) Within the migraine sample, do migraine characteristics and gender-based discrimination significantly predict mental health symptoms? and (3) Within the migraine sample, does gender-based discrimination predict migraine characteristics? We hypothesized that levels of anxiety, depressive, and trauma symptoms along with gender-based discrimination would be higher within the migraine versus non-migraine group. Further, we hypothesized that migraine frequency, migraine-related disability, and gender-based discrimination would positively predict levels of anxiety, depressive, and trauma symptoms for the migraine sample. Lastly, we predicted that gender-based discrimination would positively predict migraine frequency and migraine-related disability for the migraine sample.

## Methods

### Participants

The purpose of the study was to examine the relationship between migraine, mental health outcomes, and gender-based discrimination among adult women.

### Measures

#### Eligibility Screener

The eligibility screener was designed to assess participant eligibility at the beginning of the survey. Participants were asked (a) Are you at least 18 years of age? and (b) Do you identify as a woman? Participants who answered “yes” to each question proceeded to the participant consent form. Participants who answered “no” to one or all questions were discontinued from the study.

#### Migraine Characteristics

Participants within the migraine group were asked to report their migraine characteristics. Participants completed the Pre-Treatment Migraine Headache Questionnaire to characterize their migraine frequency (Massachusetts General Hospital, n.d.), and the Migraine Disability Assessment Test (MIDAS; AstraZeneca Pharmaceuticals, 2007 to assess how their lives are affected by their migraine and characterize their migraine-related disability. We included the entire versions of the Pre-Treatment Migraine Headache Questionnaire and the MIDAS, however only analyzed responses of relevance to our research questions. Using this scale, participants were asked to numerically report how frequently their migraines have interfered within their daily life in the past 3 months, answering questions such as “On how many days in the last 3 months did you miss work or school because of your headaches?” Prior examinations of the psychometric properties of the MIDAS have reported excellent test-retest reliability of approximately *r* = 0.8 and internal consistency of α = 0.76 in United States samples (Stewart et al., 2001).

#### Mental Health Symptoms

Participants reported their anxiety, depressive, and trauma symptoms. The PTSD Checklist for DSM-5 (PCL-5; Weathers et al., 2013) was used to assess issues (e.g. troubles sleeping, negative feelings, memory difficulties, etc.) participants may experience in response to overtly stressful life events and to measure their possible experiences with trauma. The PCL-5 consists of 20 questions which are rated on a 5-point Likert scale ranging from 0 (i.e. “Not at all”) to 4 (i.e. “Extremely”) with final scores ranging from 0 to 80 (Weathers et al., 2013). The PCL-5 has been found to show strong internal consistency (Cronbach α = 0.94) and test-retest reliability (*r* = 0.82; Blevins et al., 2015). While there is currently no clinical cut off for the PCL-5, the National Center for PTSD suggests that a total score of 31–33 indicates the individual may be experiencing clinical levels of PTSD symptoms (Blevins et al., 2015; U.S. Department of Veterans Affairs, n.d.). The Patient Health Questionnaire-9 (PHQ-9; Kroenke et al., 2001) was used to measure participants’ possible depressive symptoms. The PHQ-9 includes 9-items that participants can rate from 0 (i.e. “Not at all”) to 3 (i.e. “Nearly every day”). The PHQ-9 has demonstrated excellent internal reliability with a Cronbach’s α of 0.89 and test-retest reliability of *r* = 0.84 (Kroenke et al., 2001). The recommended clinical cut off for PHQ-9 is a score of 10, however scores can range from 8 to 11 (Manea et al., 2012). The Generalized Anxiety Disorder 7-Item Scale (GAD-7; Spitzer et al., 2006) was used to assess participant’s levels of anxiety-related symptoms. The GAD-7 consists of 7 items rated on a 4-point Likert scale from 0 (i.e. “Not at all”) to 3 (“Nearly every day”). The GAD-7 has exhibited excellent internal consistency (Cronbach α = 0.92) and good test-retest reliability (intraclass correlation = 0.83; Spitzer et al., 2006). The clinical cut off for the GAD-7 is a score of 10 or higher (Spitzer et al., 2006).

#### Gender-Based Discrimination

Participants’ experiences with discrimination were measured using the Intersectional Discrimination Index (InDI; Scheim and Bauer, 2019). An adapted version of the InDI was used to specifically assess participant’s experiences with gender-based discrimination. Consequently, the “Because of who you are” statements were adjusted to read as “Because you are a woman” statements. This scale measures three areas of discrimination (i.e. anticipated discrimination (InDI-A), lifetime day-to-day discrimination (InDI-D), and major discrimination (InDI-M)). Major discrimination refers to the denial of experiences, versus inferior experiences, based on aspects of an individual’s identity. The InDI-A features nine items rated on a 5-point scale ranging from 0 (i.e. “Strongly disagree”) to 4 (i.e. “Strongly agree”). Total scores ranged from 0 to 36, with higher scores indicating worse anticipated discrimination. The InDI-D consists of nine items rated on a 3-point scale with the options “Never” (i.e. scored 0), “Yes, but not in the past year” (i.e. scored 0), “Yes, once or twice in the past year” (i.e. scored 1), or “Yes, many times in the past year” (i.e. scored 2). The InDI-M was excluded due to the extended length of the measure and restricted time frame of the project. All three sections of the InDI have demonstrated excellent construct validity and test-retest reliability, InDI-A (0.72), InDI-D (0.70), InDI-M (0.72; Scheim and Bauer, 2019). For the InDI, the recommended cut point to indicate psychological distress is a score of 13 or higher (Scheim and Bauer, 2019).

#### Educational Debriefing

After completing the survey, participants were redirected to an educational debriefing form which explained the purpose of the study and provided participants with a list of mental health resources.

### Procedure

Survey responses were collected from November 2023 to December 2023. Participants were recruited via the University of Regina’s Psychology Participant Pool. Participants were assessed for eligibility through an online survey. Upon being deemed eligible, all participants provided informed electronic consent, reported if they experienced migraine and were subsequently assigned to either migraine, or non-migraine group, and completed the online survey. Participants in the migraine group completed measures regarding their migraine experiences. The study was approved by the University of Regina’s Research Ethics Board (#2023-369).

### Data analysis

Descriptive statistics (i.e. means, standard deviations, and frequencies) were calculated for the demographic variables. Total scores were calculated for the PCL-5, PHQ-9, GAD-7, InDI-A, and InDI-D. To test the hypotheses, a series of independent samples *t*-tests and hierarchical multiple regressions were performed on IBM Statistical Package for Social Sciences Version 27 (SPSS; IBM Corp, 2020).

## Results

### Internal consistency

All scales and subscales demonstrated good (i.e. α > 0.80) to great (i.e. α > 0.90) reliability. GAD-7 (α = 0.899), PHQ-9 (α = 0.890), PCL-5 (α = 0.949), InDI-A (α = 0.912), InDI-D (α = 0.904) all demonstrated good to great reliability and were therefore included in the analysis.

### Demographic information

A sample of adult women (*N* = 292) participated in this case-control study. Within the sample, *n* = 110 participants identified as living with migraine and *n* = 182 indicated they did not experience migraine. All participants were enrolled in a 100- or 200-level psychology course and were between the ages of 18 and 61 years, though the study primarily consisted of young adults (i.e. 18–26 years-old; 79.74%). On average, participants self-identified straight (77.05%) and white (47.60%). More detailed demographic characteristics of each sample are presented in Table 1.

**Table 1.** Participant demographics.

Group	Migraine (*n* = 110)	Non-migraine (*n* = 182)
Variable	*M* (SD)	*M* (SD)
Age in years	24.03 (8.30)	21.74 (5.79)
	*N* (%)	*N* (%)
Sexual orientation		
Straight	81 (73.63%)	144 (79.12%)
Lesbian	___	2 (1.09%)
Bisexual	18 (16.36%)	11 (6.04%)
Queer	1 (0.90%)	6 (3.29%)
Pansexual	3 (2.72%)	4 (2.19%)
Asexual	___	3 (1.64%)
Other	4 (3.63%)	7 (3.84%)
Race		
White	62 (55.45%)	77 (42.30%)
Black	5 (4.54%)	27 (14.83%)
Indigenous	4 (3.63%)	9 (4.94%)
Chinese	2 (1.81%)	2 (1.09%)
Filipino	2 (1.81%)	5 (2.74%)
South Asian	14 (12.72%)	19 (10.43%)
Arab	___	2 (1.09%)
Other	5 (4.54%)	14 (7.69%)

*N* = 292.

### Mental Health symptoms and discrimination experiences

To test if mental health symptoms and experiences of gender-based discrimination differed significantly between the migraine and non-migraine sample, a series of independent samples *t*-tests were conducted. Results indicated worse anxiety scores (*t* (286) 5.190, *p* ≤ 0.001, one-tailed), depression scores (*t* (267) 4.276, *p* ≤ 0.001, one-tailed), and trauma scores (*t* (263) 4.457, *p* ≤ .001, one-tailed) among the migraine group compared to the non-migraine group. The effect sizes for anxiety (Cohen’s *d* = 0.632), depression (Cohen’s *d* = 0.538), and trauma (Cohen’s *d* = 0.570) scores were all in the medium range. Furthermore, a series of independent samples *t*-tests indicated significantly greater levels of anticipated discrimination (*t* (268) 2.225, *p* = 0.013, one-tailed) and lifetime day-to-day discrimination (*t* (196.077) 2.692, *p* = 0.004, one-tailed) in the migraine group compared to the non-migraine group. Effect sizes for anticipated discrimination (Cohen’s *d* = 0.280) and lifetime day-to-day discrimination (Cohen’s *d* = 0.346) scores were the medium range. Independent samples *t*-test results are shown in Table 2.

**Table 2.** Independent samples *t*-tests results.

Variable	Migraine	Non-migraine	*p*
	*M* (SD)	*M* (SD)	
Anxiety	19.88 (5.70)	16.53 (5.04)	<0.001
Depression	22.73 (6.69)	19.36 (5.98)	<0.001
Trauma	56.54 (19.75)	46.27 (16.97)	<0.001
Anticipated discrimination	22.84 (8.82)	20.41 (8.57)	0.013
Lifetime day-to-day discrimination	25.27 (8.95)	22.33 (8.20)	0.004

*N* = 292.

### Correlations

To determine which demographic variables to include as control variables within the series of hierarchical multiple regressions, bivariate correlations between the demographic characteristics and outcome variables in the migraine group were conducted. Sexual orientation was found to be significantly correlated with trauma (*p* = 0.010), so it was included as a control variable in subsequent analyses.

### Migraine frequency and migraine-related disability

To assess if migraine characteristics significantly predicted mental health symptoms, a series of hierarchical multiple regressions were conducted (see Table 3). Trauma, anxiety, and depression symptoms served as the dependent variables in the first, second, and third multiple regression analysis, respectively. Sexual orientation and migraine characteristics (i.e. migraine frequency and migraine-related disability) were entered as the predictor variables. For trauma symptoms, both sexual orientation and migraine characteristics were significant predictors. Furthermore, the change from the first model was statistically significant. The analysis also indicated that sexual orientation was not a significant predictor of anxiety symptoms; however, migraine characteristics was a significant predictor of anxiety symptoms. Additionally, the change from the first model to the second model was not statistically significant. Finally, it was found that migraine characteristics, but not sexual orientation, were significant predictors of depression symptoms. The change from the first to second model was statistically significant. The last model accounted for 29.7% (*R* ^2^ = 0.297) of the variance in trauma symptoms.

**Table 3.** Hierarchical multiple regression analyses predicting trauma symptoms.

Dependent variable	Model	*N*	*F*	*p*	*R* ^2^	Δ*F*	Δ*R* ^2^	Predictor variable	β	*t*	*p*
Trauma symptoms	1	107	5.780	0.019	0.072			Sexual orientation	2.893	2.404	0.019*
	2	96	3.599	0.002	0.297	3.124	0.226	Migraine characteristics	0.325	2.680	0.009* *
Anxiety symptoms	1	107	2.512	0.117	0.032			Sexual orientation			
	2	108	2.215	0.036	0.202	2.135	0.170	Migraine characteristics	0.100	2.718	0.008* *
Depression symptoms	1	107	2.302	0.133	0.029			Sexual orientation			
	2	101	3.818	0.001	0.304	2.946	0.275	Migraine characteristics	0.133	3.287	0.002* *

*N* = 292.

* *p* < 0.05. **p < 0.01.

### Gender based discrimination and mental health symptoms

A second series of hierarchical multiple regression analyses were conducted to determine if gender-based discrimination significantly predicted mental health symptoms within the migraine group (see Table 4). Depression, anxiety, and trauma symptoms were entered as the dependent variables for the first, second, and third multiple regression analysis, respectively. Sexual orientation and gender-based discrimination (i.e. anticipated discrimination and lifetime day-to-day discrimination) were entered as the predictor variables. For depressive symptoms, sexual orientation was not significant, but gender-based discrimination was a significant predictor. The change from the first to second model was also statistically significant. In model two, lifetime day-to-day discrimination was a significant predictor of depressive symptoms. The final model accounted for 36.6% (*R* ^2^ = 0.366) of the variance in depressive symptoms. Sexual orientation was found to not be a significant predictor of anxiety symptoms; however, gender-based discrimination significantly predicted anxiety symptoms. Additionally, the change from the first model to the second model using anxiety symptoms as the dependent variable was statistically significant. The final model accounted for 29.7% (*R* ^2^ = 0.297) of the variance in anxiety symptoms. Both sexual orientation and gender-based discrimination were found to be predictive of trauma symptoms. The change from the first to second model was significant. Furthermore, in model two, anticipated discrimination and lifetime day-to-day discrimination were significant predictors of trauma. The final model accounted for 64.2% (*R* ^2^ = 0.642) of the variance in trauma.

**Table 4.** Hierarchical multiple regression analysis of gender-based discrimination and mental health symptoms.

Dependent variable	Model	*N*	*F*	*p*	*R* ^2^	Δ*F*	Δ*R* ^2^	Predictor	β	*t*	*p*
Depression symptoms	1	107	2.781	0.099	0.029						
	2	101	17.526	<0.001	0.366	24.204	0.337	Lifetime day-to-day discrimination	0.426	5.921	<0.001
Anxiety symptoms	1	107	3.067	0.083	0.032						
	2	101		<0.001	0.297	17.352	0.265	Lifetime day-to-day Discrimination	0.273	4.254	<0.001
Trauma symptoms	1	107	6.859	0.010	0.072			Sexual orientation	2.893	2.619	0.010
	2	101	20.294	<0.001	0.642	25.150	0.340	Anticipated discrimination	0.469	2.171	0.033
								Lifetime day-to-day discrimination	1.060	5.073	<0.001

*N* = 292.

### Gender based discrimination and migraine characteristics

A third series of hierarchical multiple regression analyses were conducted to determine if gender-based discrimination significantly predicted migraine characteristics within the migraine group (see Table 5). Migraine frequency, migraine-related absence from work or school, migraine-related decrease in work or school productivity, migraine-related absence in housework, migraine-related reduction in housework, and migraine-related absence from social events were entered as the dependent variables in the first, second, third, fourth, and fifth multiple regression analysis, respectively. Sexual orientation and gender-based discrimination were entered as the predictor variables. Results indicated that neither sexual orientation nor gender-based discrimination were predictive of migraine frequency; however, in the second model, lifetime day-to-day discrimination was a significant predictor of migraine frequency. The final model accounted for 5.1% (*R* ^2^ = 0.051) of the variance in migraine frequency. For migraine disability (i.e. migraine-related absence from work or school), neither sexual orientation nor gender-discrimination were found to be significant predictors. The second model accounted for 4.6% (*R* ^2^ = 0.046) of the variance in migraine-related absence from work or school. In the third model, with migraine disability (i.e. migraine-related decrease in work or school productivity) as the dependent variable, sexual orientation was not found to be a predictor; however, gender-based discrimination was a predictor of migraine disability. Additionally, the change from the first to the second model was statistically significant. In the second model, lifetime day-to-day discrimination was a significant predictor of migraine-related decrease in work or school productivity. The last model accounted for 33.8% (*R* ^2^ = 0.338) of the variance in migraine-related decrease in work or school productivity. For migraine disability measures by migraine-related absence in housework, gender-based discrimination, but not sexual orientation, was found to be a significant predictor. The change from the first to second model was statistically significant. For the second model, lifetime day-to-day discrimination was a significant predictor of migraine-related absence in housework. The final model accounted for 33.7% (*R* ^2^ = 0.337) of the variance in migraine-related absence in housework. The regression using migraine-related reduction in housework as the dependent variable found that gender-based discrimination but not sexual orientation was a predictor of migraine-related reduction in housework. The change from the first to second model was statistically significant. In model two, lifetime day-to-day discrimination was a significant predictor of migraine-related reduction in housework. The final model accounted for 1.0% (*R* ^2^ = 0.010) of the variance in migraine-related reduction in housework. Lastly, the final regression using migraine-related absence from social events as the dependent variable found only gender-based discrimination but not sexual orientation to be a significant predictor. The change from the first to second model was not significant. In model one, sexual orientation was a significant predictor of migraine-related absence from social events. The final model accounted for 34.6% (*R* ^2^ = 0.346) of the variance in migraine-related absence from social events.

**Table 5.** Hierarchical multiple regression analysis of gender-based discrimination and migraine characteristics.

Dependent variable	Model	*N*	*F*	*p*	*R* ^2^	Δ*F*	Δ*R*^2^	Predictor	β	*t*	*p*
Migraine frequency	1	107	0.153	0.697	0.002						
	2	101	35.746	0.194	0.051	2.327	0.049	Lifetime day-to-day discrimination	0.132	2.091	0.039
Migraine-related absence from work or school	1	107	0.327	0.569	0.064						
	2	101	1.246	0.299	0.046	1.703	0.042				
Migraine-related decrease in work or school productivity	1	107	0.229	0.633	0.054						
	2	101	3.307	0.025	0.338	4.834	0.111	Lifetime day-to-day discrimination	0.492	2.952	0.004.
Migraine-related absence in housework	1	107	0.072	0.789	0.030						
	2	101	3.247	0.026	0.337	4.831	0.113	Lifetime day-to-day discrimination	0.289	2.918	0.005
Migraine-related reduction in housework	1	107	0.009	0.926	0.444						
	2	101	6.299	<0.001	0.010	9.443	0.197	Lifetime day-to-day discrimination	0.725	4.012	<0.001
Migraine-related absence from social events	1	107	4.261	0.042	0.226			Sexual orientation	0.658	2.064	0.042
	2	101	3.482	<0.020	0.346	2.985	0.068				

*N* = 292.

## Discussion

The current study aimed to investigate the relationship between migraine characteristics (i.e. frequency, disability), gender-based discrimination (i.e. anticipated discrimination, lifetime day-to-day discrimination), and mental health symptoms (i.e. depression, anxiety, trauma) among adult women. Results from the current study indicate that the women who experience migraine endorse more severe mental health symptoms compared to those without migraine. This finding coincides with previous studies which have found mental health symptoms to be more prevalent among individuals living with migraine than those without (de Leeuw et al., 2005; Fuller-Thomson et al., 2017; McWilliams et al., 2004; Molgat and Patten, 2005). Furthermore, experiences of gender-based discrimination were more frequently reported by the migraine group compared to the non-migraine group. Specifically, both anticipated discrimination and lifetime day-to-day discrimination scores were higher within the migraine group versus the non-migraine group. This result is consistent with previous research, which has found that women and those who live with migraine tend to more commonly report having experienced discrimination than men and those who live without migraine, respectively (Klonoff and Landrine, 1995; Rosendale et al., 2022; Statistics Canada, 2023). However, little research has been conducted on the specific role of discrimination in the experiences of women who are also living with migraine.

Among the migraine group, results indicated that migraine frequency was a significant predictor for anxiety, depression, and trauma symptoms. These findings support previous research which demonstrates that more frequent migraines often lead to greater psychiatric symptoms (Minen et al., 2016a; Zwart et al., 2003). Further, within the migraine group, gender-based discrimination positively predicted levels of anxiety, depression, and trauma. Specifically, lifetime day-to-day discrimination was significantly predictive of depression, anxiety, and trauma symptoms, while anticipated discrimination was only a significant predictor for symptoms of trauma. These findings are consistent with previous research which demonstrates that women who experience gender-based discrimination typically have worse mental health symptoms (Calabrese et al., 2015; Fischer and Holz, 2010; Hackett et al., 2019). Further, individuals who have experienced traumatic event(s) often report significant levels of anxiety surrounding future traumatic events (Center for Substance Abuse Treatment (United States), 2014). Consequently, the higher level of prospective anxiety endured among individuals with PTSD may explain the stronger association between anticipated discrimination and trauma over the other mental health symptoms.

Gender-based discrimination and, specifically, lifetime day-to-day discrimination, was a significant predictor of migraine frequency, migraine-related decrease in work or school productivity and reduction in housework. However, lifetime day-to-day discrimination was not significantly predictive of migraine-related absence in work or school or absence from social events. Furthermore, anticipated discrimination was not significantly predictive of migraine frequency or migraine-related disability. Current literature suggests that individuals who experience higher levels of discrimination may see negative impacts to their health (American Psychological Association, 2019; Earnshaw et al., 2016; Hua et al., 2023). Therefore, it is anticipated that lifetime day-to-day discrimination would have a significant impact on individuals’ migraine characteristics. A study investigating the relationship between anticipated discrimination, experienced discrimination, and symptoms of depression found experienced discrimination to be associated with worse long-term depressive symptoms (Jhon et al., 2021). Consequently, it is possible that the stress of experiencing gendered discrimination (i.e. lifetime day-to-day discrimination), rather than thinking about potential discrimination (i.e. anticipated discrimination), may have a greater effect on long-term physical health outcomes (e.g. migraine symptoms). However, due to the limited research on gender-based discrimination among individuals living with migraine, the differential effects of types of discrimination on migraine-related disability are difficult to identify.

Findings from this study contribute to the understanding of the relationship between mental health symptoms and migraine among adult women. Current research suggests that mental health symptoms (i.e. anxiety, depression, trauma) are comorbid with migraine (de Leeuw et al., 2005; Fuller-Thomson et al., 2017; McWilliams et al., 2004; Molgat and Patten, 2005). The current study aligns with these findings, suggesting mental health symptoms are more severe among individuals living with migraine. Additionally, experiences of discrimination have been commonly found among both women and migraine samples (Rosendale et al., 2022; Statistics Canada, 2023). However, the role that discrimination, generally, and gender-based discrimination, specifically, plays on migraine characteristics is greatly understudied. Adding to emergent literature, the current study suggests that gender-based discrimination plays a role in mental health symptoms and migraine-related disability among individuals living with migraine. Current literature suggests that many healthcare professionals believe that they are uneducated and/or inexperienced in providing proper treatment and management for migraine generally (Minen et al., 2016b) and among women (Verhaak et al., 2021). Therefore, considering the current study’s findings, adjusting the medical treatment of migraine to target women and individuals who have experienced gender-based discrimination may significantly improve the lives of those living with migraine. Research further suggests that healthcare providers currently report lacking in-depth knowledge surrounding non-pharmaceutical migraine interventions (Minen et al., 2016b; Verhaak et al., 2021). The comorbidity of mental health symptoms and migraine (de Leeuw et al., 2005; Fuller-Thomson et al., 2017; McWilliams et al., 2004; Molgat and Patten, 2005) highlights a pressing need to update the current healthcare curriculum to include comprehensive teachings surrounding psychological-based treatment of migraine (Minen et al., 2016b; Verhaak et al., 2021). Lastly, the findings provide direction for future research which further investigates the unique relationship between mental health symptoms, discriminatory experiences, and migraine.

### Limitations and directions for future research

To our knowledge, this study is one of the first to investigate gender-based discrimination among adult women experiencing migraine. Consequently, the present findings add to a growing area of literature and provide support for other researchers to further pursue work within this area. When interpreting these findings, it is important to consider the current study’s limitations. For example, as the data was gathered using the University of Regina’s Psychology Participant Pool, all participants were students registered in 100- or 200-level psychology courses, resulting in most participants being predominantly young adults (i.e. 18–26 years of age), and therefore limiting the generalizability of the findings. It should also be noted that most of the participants self-identified their racial background as White and their sexual orientation as straight, further limiting the generalizability of the findings. Furthermore, participants were not asked if they have a medical diagnosis of migraine in order to participate in the current study as individuals who suffer from migraine often go undiagnosed. As migraine and other forms of headaches can be difficult to distinguish, the findings of the current study may have a reduced specificity and validity as participants may experience a variety of headache types, rather than strictly migraine.

Nevertheless, the present study had a sufficient sample size (i.e. 292) of both women living with (i.e. 110) and without migraine (i.e. 182), therefore increasing the generalizability and accuracy of the results (Andrade, 2020; Faul et al., 2009). In addition, as the surveys were administered via online self-report measures, the effects of social desirability and response biases among participants may have affected the results (Salters-Pedneault, 2023; Wright, 2005), so future studies should control for these biases (i.e. use of indirect questioning techniques; Meisters et al., 2020). Future research should explore whether women who are also living with migraine experience more frequent gender-based discrimination than women who are not living with migraine or men who are living with migraine. Moreover, more research is still needed to better understand the intersectional relationship between mental health symptoms, discriminatory experiences, and migraine characteristics among women. That is, considering that lifetime day-to-day discrimination was more frequently found to be predictive of mental health symptoms and migraine-related disability, future studies should investigate the effect that different discrimination experiences (e.g. ableism, racism) have on migraine characteristics. Within future work, longitudinal study designs may offer more insight toward the consistency and severity of the role between mental health and migraines. In addition, only individuals who self-identified as women were included within the sample. Consequently, future research may investigate these outcomes within a sample of individuals whose sex assigned at birth is female, since both biological and social factors can influence one’s overall health outcomes and pain experiences.

### Conclusion

The present study expands the current body of existing research literature investigating mental health symptoms among individuals experiencing migraine. Specifically, it explores the impact of migraine characteristics and gendered discrimination on anxiety, depression, and trauma symptoms among women living with migraine. The results of the current study support existing literature suggesting that mental health symptoms are more severe among migraine populations compared to the general population (de Leeuw et al., 2005; Fuller-Thomson et al., 2017; McWilliams et al., 2004; Molgat and Patten, 2005). Moreover, the present findings support previous literature indicating that individuals with migraine have a history of discriminatory experiences (Rosendale et al., 2022). The results of the current study add to an emergent field of literature suggesting that gender-based discrimination influences mental health symptoms and migraine-related disability among women living with migraine. Specifically, findings from this study demonstrate that gender-based discrimination plays a role in work or school productivity and household work among women living with migraine. Therefore, consideration of this study’s results may allow for mental health and migraine assessments and treatments that are tailored specifically toward women living with migraine, due to greater awareness of the experience of those living with migraine.
